# Preferences for HIV pre‐exposure prophylaxis formulations and delivery among young African women: results of a discrete choice experiment

**DOI:** 10.1002/jia2.26422

**Published:** 2025-02-16

**Authors:** Wendy W. Dlamini, Brenda G. Mirembe, Meighan L. Krows, Sue Peacock, Philip L. Kotze, Pearl Selepe, Jenni Smit, Nelly Mandona, Cheryl Louw, Harriet Nuwagaba‐Biribonwoha, Victor O. Omollo, Zinhle Zwane, Ravindre Panchia, Noluthando Mwelase, Melissa Senne, Logashvari Naidoo, Rachel Chihana, Sinead Delany‐Moretlwe, Katherine Gill, Pippa MacDonald, Alastair van Heerden, Shannon Bosman, Remco P. H. Peters, Philip du Preez, Amy Ward, Connie Celum, Renee Heffron, Jennifer Velloza, for the INSIGHT Study Team

**Affiliations:** ^1^ Department of Epidemiology University of Washington Seattle Washington USA; ^2^ Makerere University‐Johns Hopkins University Research Collaboration Kampala Uganda; ^3^ Department of Global Health University of Washington Seattle Washington USA; ^4^ Qhakaza Mbokodo Research Clinic Ladysmith South Africa; ^5^ Aurum Klerksdorp Clinical Research Centre Klerksdorp South Africa; ^6^ Wits Maternal Adolescent and Child Health Research Department of Obstetrics and Gynaecology University of the Witwatersrand Durban South Africa; ^7^ University of North Carolina Global Projects‐Kamwala Health Centre Kamwala Zambia; ^8^ Madibeng Centre for Research Brits South Africa; ^9^ ICAP Eswatini Prevention Center Mbabane Eswatini; ^10^ Kenya Medical Research Institute Kisumu Kenya; ^11^ Setshaba Research Centre Pretoria South Africa; ^12^ Perinatal HIV Research Unit University of the Witwatersrand Soweto South Africa; ^13^ University of the Witwatersrand Johannesburg South Africa; ^14^ SAMRC Chatsworth Clinical Research Site Chatsworth South Africa; ^15^ Kamuzu University of Health Sciences – Johns Hopkins Research Project Blantyre Malawi; ^16^ Wits RHI University of the Witwatersrand Johannesburg South Africa; ^17^ Desmond Tutu HIV Centre Cape Town South Africa; ^18^ Center for Community‐Based Research Human Sciences Research Council Pretoria South Africa; ^19^ Foundation for Professional Development Research Unit East London South Africa; ^20^ Vuka Research Clinic University of Cape Town Cape Town South Africa; ^21^ Department of Medicine University of Washington Seattle Washington USA; ^22^ Department of Medicine University of Alabama at Birmingham Birmingham Alabama USA; ^23^ Department of Epidemiology & Biostatistics University of California San Francisco San Francisco California USA

**Keywords:** discrete choice experiment, HIV prevention, long‐acting, pre‐exposure prophylaxis, preferences, trade‐offs

## Abstract

**Introduction:**

Oral HIV pre‐exposure prophylaxis (PrEP) is highly effective, but adherence is challenging for young women. Products centred around women's preferences could address adherence barriers. Using a longitudinal discrete choice experiment (DCE), we examined young African women's preferences around PrEP product formulation and delivery attributes before and after initiating oral PrEP.

**Methods:**

We enrolled HIV‐negative women from six African countries in a prospective cohort from August 2022 to June 2023. Women completed two DCEs on PrEP products and PrEP delivery. At enrolment and month 1, participants completed the DCE about PrEP products with 16 randomly assorted choice sets assessing product form and dosing, dose forgiveness, drug reversibility, weight change and antiretroviral or immune‐based mechanism attributes. At month 3, participants completed the DCE about PrEP delivery evaluating preferences related to location to collect doses, packaging, product storage, type of HIV test and costs. Preference weights (PW) were estimated with a hierarchical Bayesian model; higher positive numbers indicate greater preference for an attribute. Importance scores compare relative importance across the five attributes; higher scores indicate greater importance.

**Results:**

Two thousand eight hundred and forty‐seven women completed enrolment and month 1 DCEs; the median age was 24 years (range: 16–30) and 92.8% initiated daily oral PrEP. Product form and dosing was the most important attribute at enrolment and month 1. At enrolment, women preferred small oral pills taken monthly (preference weight [PW]: 0.67; 95% confidence interval [CI]: 0.58−0.77), and at month 1, they preferred a 6‐monthly injection (PW: 0.56; 95% CI: 0.46−0.65). In the month 3 DCE, location was the most important PrEP delivery attribute with a strong preference for a youth‐friendly or non‐governmental organization (PW: 0.25; 95% CI: 0.19−0.30) or health facility (PW: 0.21; 95% CI: 0.17−0.25); mobile clinic or van was least preferred. The cost of the product was the second most important product delivery attribute.

**Conclusions:**

Young African women preferred discreet, less frequently administered PrEP formulations, particularly after 1 month of taking daily oral PrEP. Long‐acting formulations are needed to meet women's preferences. Coupled with the preferred PrEP delivery location and cost, the highlighted PrEP product characteristics have the potential to increase PrEP uptake.

## 1 INTRODUCTION

Young African women accounted for 63% of new HIV acquisitions in 2021 [1]. Daily oral tenofovir‐based pre‐exposure prophylaxis (PrEP) is a highly effective HIV prevention approach [2, 3], but adherence is challenging for young women. Multiple demonstration projects have found high PrEP uptake among young women but <20% persist and adhere through 12 months [4, 5]. Barriers to PrEP adherence include forgetting to take the pill, large tablet size, side effects and stigma [6]. Structural barriers include long distances to health facilities, transportation costs and lacking bottle storage [7, 8].

Longer‐acting PrEP formulations (e.g. injectables, monthly tablets) could address some of these barriers to daily oral PrEP. Additionally, facilitating PrEP accessibility through less medicalized PrEP delivery options could meet women's needs and increase PrEP coverage [9]. The development of new PrEP formulations and implementation approaches would benefit from an understanding of users’ priorities, which can be assessed through discrete choice experiments (DCEs) that quantify consumers’ preferences around a predetermined set of characteristics [10]. DCE studies that have investigated various features of PrEP products in Africa [11, 12, 13, 14] and elsewhere [15, 16, 17] indicate that product efficacy and duration are strong determinants of choice. A review of 18 DCE studies from 13 countries globally identified dosing frequency, cost and PrEP effectiveness as primary factors influencing PrEP preferences [18]. However, these studies were generally conducted among PrEP‐naïve participants and did not assess young women's preferences for alternative PrEP delivery locations to promote access and uptake.

To learn about PrEP product and delivery preferences, and how experience with taking daily oral PrEP might shape those preferences, we conducted a longitudinal DCE to assess women's preferences about attributes of long‐acting PrEP before and 1 month after taking daily oral PrEP. We also conducted a separate DCE to assess preferences around attributes of PrEP service provision 3 months after PrEP initiation. This work was nested in a large, multi‐country cohort of young African women. The objective of this DCE was to inform planning for vaginal ring and injectable PrEP rollout and longer‐acting PrEP formulations currently in development.

## 2 METHODS

### 2.1 Study setting, participants and design

INSIGHT was a prospective open‐label study of daily oral tenofovir‐based PrEP use among young women, conducted from August 2022 to August 2023 in 20 clinical research sites: 15 in South Africa and one site each in Eswatini, Kenya, Malawi, Uganda and Zambia (ClinicalTrials.gov #NCT05746065). Eligibility criteria included being a cisgender woman, 16–30 years old, HIV negative, sexually active and interested in taking PrEP. Recruitment involved community sensitization and outreach and is described in detail elsewhere [19]. At enrolment, women were offered HIV testing and PrEP according to national guidelines. Participants with reactive HIV tests were referred for HIV treatment. PrEP initiation was not a requirement for enrolment and participants were allowed to have previously used PrEP. Follow‐up was conducted at 1, 3 and 6 months to coincide with standard PrEP refill intervals (1 month after initiation and quarterly thereafter). DCEs were conducted within the first 3 months of the study because most drop‐offs in PrEP adherence among young women happen by 3 months post‐PrEP initiation, reflecting possible changing preferences around PrEP after experience with daily pill‐taking [5].

**Figure 1:**
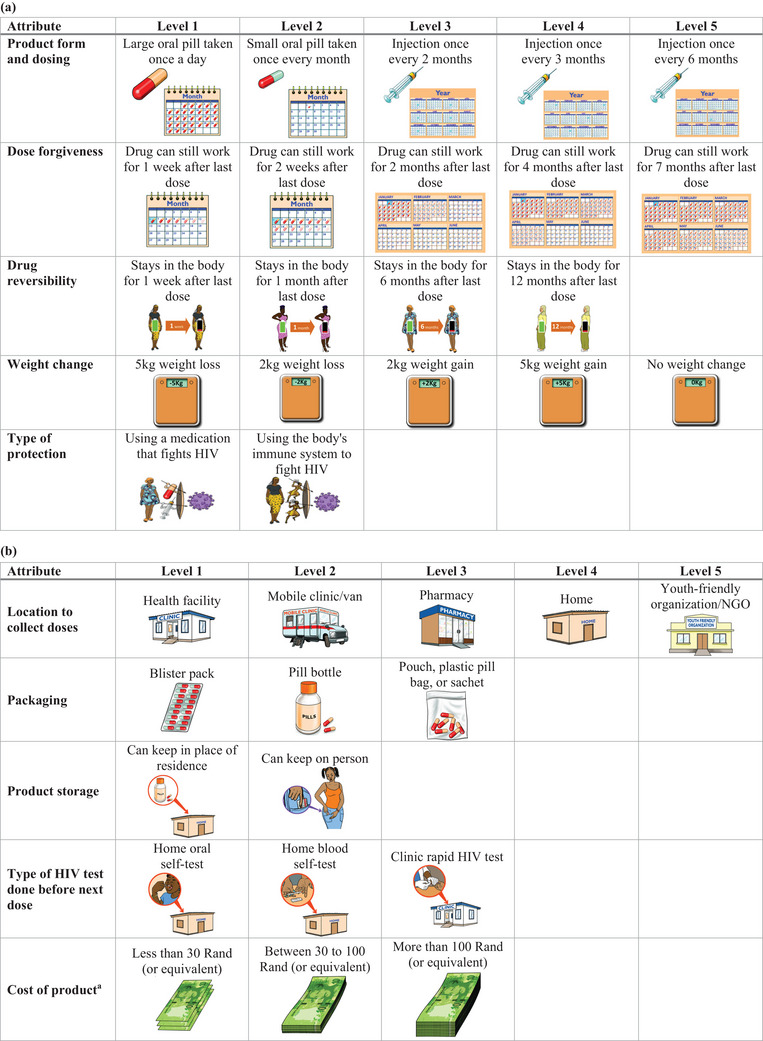
Illustrations and pictograms used in the DCE surveys. PrEP product attributes and levels assessed in DCE at enrolment and month 1 (a) and PrEP delivery attributes and levels assessed at month 3 (b). In these figures, the rows represent the attributes, and the columns, levels within each attribute. ^a^ For the “cost of product” (August 2022 conversion rate), 30 Rand (South African rand) is approximately $1.50 and 100 Rand (South African rand) is approximately $5.30 United States dollars. DCE, discrete choice experiment; kg, kilograms; NGO, non‐governmental organization; PrEP, pre‐exposure prophylaxis.

INSIGHT incorporated two DCEs—one to assess preferences around PrEP products and another on PrEP delivery. The PrEP product DCE was conducted longitudinally at enrolment and month 1 to assess whether preferences changed after at least 1 month of use of daily PrEP. It included five attributes of PrEP products: form and dosing; dose forgiveness; drug reversibility; weight change; and protection type (Figure 1a). The PrEP delivery DCE was implemented at month 3 only and attributes included: location to collect doses; PrEP product packaging; product storage options; type of HIV test to accompany refills; and cost of product (Figure 1b). The PrEP delivery DCE was conducted at month 3 to allow participants sufficient time to experience oral PrEP use before assessing their PrEP delivery preferences.

### 2.2 DCE pre‐testing

We conducted a four‐step DCE development process. First, we consulted HIV prevention researchers, young women and community advisory boards (CABs) at each site. Initial CAB discussions focused on domains young women might consider when evaluating products, resulting in one PrEP product DCE and one PrEP delivery DCE, each with 5–10 attributes. Second, initial DCEs were developed by the investigators based on these consultations and review of the literature [11, 12, 13, 14, 16, 20]. Third, we received feedback on the draft DCEs from CABs on definitions and levels of attributes which was incorporated into the final version with five attributes in each DCE. For example, prior to CAB meetings, we considered attributes related to side effects like headaches, but CAB members indicated that weight gain would be a critical side effect for young women. CAB feedback helped establish DCE protocols with standard definitions for attributes for staff to use with participants. Fourth, sites and CABs provided feedback about the pictorial representations of each attribute level. For example, the type of protection graphic initially included complex immune cell images, which we simplified during this feedback process.

### 2.3 Data collection

The DCEs were implemented using Sawtooth Software [21]. The design utilized a non‐orthogonal D‐efficient algorithm to construct a fractional factorial experimental design. We used the balanced overlap method to randomly assign choice sets. For each DCE, participants viewed 16 different choice sets representing distinct product and delivery options and were asked to choose between the sets (choice A, choice B, neither). For the PrEP product DCE, each participant saw the same 16 sets, presented in the same order, at enrolment and month 1 to determine whether product preferences changed over time with oral PrEP use.

Site staff were trained in Sawtooth and DCE administration. Research staff logged into the Sawtooth application using a tablet computer, entered the participant ID which launched the DCE for that visit, and then handed the tablet to the participant for DCE completion while remaining present to answer questions as needed. The DCE data were quality‐checked regularly to address any issues with completion and uploading.

Data on participant demographics, PrEP use, sexual behaviour, and HIV salience and risk perception were collected at enrolment, month 1 and month 3 via interviewer administration using DFdiscover 2022 software (Seattle, WA). Enrolment data was used to calculate HIV Salience and Perception (HPS) and modified VOICE HIV risk scores. The HPS tool (possible range: 9–36) was validated to assess concerns about the perceived risk of HIV among young African women [22]. Higher HPS scores indicate beliefs about higher vulnerability to HIV [22]. The modified VOICE HIV risk score, validated to predict HIV acquisition among African women, ranges from 0 to 8, with scores ≥5 associated with a greater risk of HIV acquisition [23]. Data from Sawtooth and DFdiscover were linked for analyses.

### 2.4 Sample size

Target enrolment for INSIGHT was 150 women per site (*N* = 3000 total), and a total of 3087 were enrolled. With the DCE parameters (16 choice tasks per survey, 3 alternatives per task and 2–5 levels per attribute), this sample size would provide sufficient information to determine preferences based on guidance in the literature [24, 25, 26].

### 2.5 Statistical analysis

Enrolment, month 1 and month 3 DCE responses were analysed using the Choice‐Based Conjoint Hierarchical Bayesian (CBC/HB) module to obtain PW in Sawtooth. This model accounts for the presence of within‐ and ‐between participants’ choice heterogeneity [27]. Twenty‐thousand iterations were done, and full convergence was attained. For each task, the women's choice (choice A, choice B, neither) was the dependent variable and the attribute level presented in the choice set was the independent variable. The PW, analogous to “utilities” in Sawtooth Software, indicate the relative desirability of each level within an attribute, with higher positive numbers indicating greater preference for a given attribute level and higher negative numbers indicating lower preference.

Attribute importance scores were calculated, which compare the relative importance of each attribute across each of the other four attributes assessed in the DCEs and summarize the impact of an attribute on choice, given the range of attribute levels included. For each participant, the importance score was calculated as a percentage of the total of all differences between the highest and lowest level PW for a given attribute by obtaining a set of attribute importance values that add to 100. Higher scores indicate a greater difference between the maximum and minimum PW (a marker of importance of the attribute in influencing the women's preference). In a pre‐specified exploratory analysis, PW and importance scores were stratified by age (≤21, >21 years) and site (South Africa vs. non‐South Africa).

We examined trade‐offs women were willing to make between pairs of attributes for product characteristics at enrolment and month 1 and product delivery at month 3 by calculating the difference in PW (a utility loss or gain) between attribute level pairs. We compared the differences between the five attributes: the difference in PW for levels in one attribute level pair (e.g. for product form and dosing, this might be the difference in PW between large oral pill daily and small oral pill monthly) was compared to the difference in PW for levels of another attribute (e.g. the difference between PW for no weight change and 5 kg weight loss). A high numeric difference indicated the participants’ willingness to choose one product attribute over the other one with a low numeric difference. These analyses were restricted to comparisons of real‐world PrEP product and delivery attributes that could be most helpful in guiding current PrEP implementation.

We used R version 4.2.3 [28] for descriptive statistics and graphical analyses.

### 2.6 Ethical considerations

The study protocol was reviewed and approved by ethics committees at each site. Adults (≥18 years) gave written informed consent. Assent and parental or guardian consent were obtained for minors (<18 years).

## 3 RESULTS

### 3.1 Participants characteristics

Three thousand three hundred and forty‐two women were screened, of whom 3087 were eligible and enrolled in INSIGHT. Of these, 3085 (99.9%), 2847 (92.2%) and 2594 (84.0%) completed enrolment, month 1 and month 3 DCEs, respectively. Enrolment and month 1 analyses were restricted to 2847 participants with complete data for both visits. The median age at enrolment was 24 years (interquartile range [IQR]: 21, 27); 72 (2.5%) of the participants were 16–17 years and 786 (27.6%) were 18–21 years. A total of 2120 (74.5%) women were from South Africa, 2641 (92.8%) initiated PrEP at enrolment and 389 (13.7%) had previously used PrEP (Table 1).

**Table 1:** Baseline characteristics of INSIGHT cohort^a^ study participants who were included in the DCE analysis

Characteristic	All participants (*N* = 2847) *n* (%)
Age, years (Median [IQR])	24.0 (21.0, 27.0)
Country	
South Africa	2120 (74.5)
Eswatini	140 (4.9)
Kenya	146 (5.1)
Malawi	149 (5.2)
Uganda	142 (5.0)
Zambia	150 (5.3)
Relationship status	
Married^b^	259 (9.1)
Partnered, not married	2484 (87.2)
Single	104 (3.7)
Education	
No schooling	62 (2.2)
Primary	210 (7.4)
Secondary or higher	2575 (90.4)
Own source of income	653 (22.9)
Employment status	
Employed^c^	491 (17.2)
Unemployed	1802 (63.3)
Student	482 (16.9)
Other	72 (2.5)
Financial support from husband or partner	2024 (71.1)
Living arrangement	
Live with parents	1314 (46.2)
Live with partner or husband	383 (13.5)
Live alone	125 (4.4)
Live with other family members	972 (34.1)
Live with friend or other living arrangement	53 (1.9)
Previously used PrEP	389 (13.7)
PrEP use at INSIGHT enrolment	
Newly initiated at enrolment	2641 (92.8)
On PrEP at enrolment	75 (2.6)
Declined	131 (4.6)
Number of sex partners, past 3 months (Median [IQR])	1.0 (1.0, 2.0)
Used condom at the last sex act	698 (24.5)
Current contraceptive method^d^	
DMPA and NET‐EN injectables	934 (45.4)
Male or female condom	399 (19.4)
Implant	481 (23.4)
Oral contraceptive	143 (7.0)
Other	100 (4.9)
HIV risk perception score (Median [IQR])^e^	24.0 (21.0, 25.0)
Modified VOICE HIV risk score (Median [IQR])^f^	5.0 (4.0, 7.0)

Abbreviations: DCE, discrete choice experiment; DMPA, depot‐medroxyprogesterone acetate; IQR, interquartile range; NET‐EN, norethisterone enanthate; PrEP, pre‐exposure prophylaxis; SD, standard deviation; VOICE, Vaginal and Oral Interventions to Control the Epidemic.

^a^ INSIGHT: Insights to advance PrEP discovery and delivery for African women.

^b^ Monogamous or polygamous marriage.

^c^ Formal and informal employment.

^d^ Numbers exclude participants with missing data on contraceptive use.

^e^ Nine question items scored 1–4 assessing women's HIV Salience and Perception (HPS). Maximum score of 36 and higher sum scores indicate greater risk perception [22].

^f^ HIV risk score based on the following: age, married/living with a partner, partner provides financial or material support, partner has other partners and alcohol use [23]. Scores ≥5 associated with an HIV incidence of >5 per 100 person‐years in validation studies of women from southern Africa in HIV prevention trials.

### 3.2 Enrolment and month 1 PrEP product DCE

**Figure 2:**
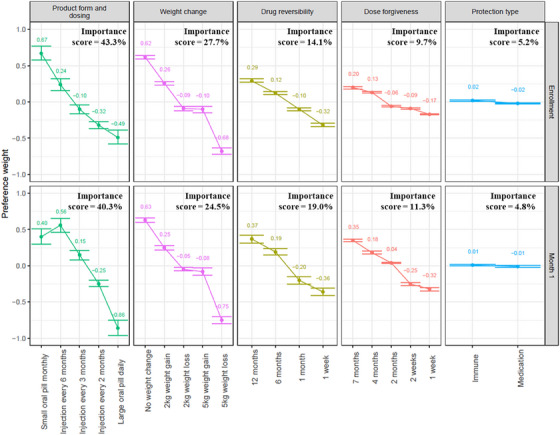
Enrolment and month 1 preference weights with 95% confidence intervals. kg, kilograms.

At enrolment and month 1 (Figure 2, Table S1, Figure S1), the product form and dosing attribute exerted the greatest influence on product choice (importance scores: 43.3% and 40.3% at enrolment and month 1, respectively). At enrolment, women most preferred small oral pills taken monthly (PW: 0.67, 95% confidence interval [CI]: 0.58, 0.77). At month 1, women most preferred an injection every 6 months (PW: 0.56, 95% CI: 0.46, 0.65). A large oral pill taken daily was least preferred at both enrolment and month 1.

The attribute related to weight change attribute was the second most influential product attribute (importance scores: 27.7% at enrolment and 24.5% at month 1) with a strong preference for no weight change with PrEP (enrolment PW: 0.62, 95% CI: 0.59, 0.64 and month 1 PW: 0.63, 95% CI: 0.60, 0.66). The least preferred weight change level was a 5 kg weight loss. Preference for the attributes related to drug reversibility (importance scores: 14.1% at enrolment and 19.0% at month 1) and dose forgiveness (importance scores: 9.7% at enrolment and 11.3% at month 1) generally followed an ordering in which longer time intervals were preferred. Protection type from an antiretroviral medication compared to an immune‐based method (e.g. neutralizing antibodies) was the least important attribute with importance scores of 5.2% at enrolment and 4.8% at month 1. Of all participants, 8.4% at enrolment and 8.2% at month 1 chose the “none” option (i.e. “I would not choose either of these HIV prevention products”) for at least one of the 16 choice sets presented. In addition, 156 (5.5%) chose none for at least half of the choice sets, of whom seven also declined PrEP at enrolment. One participant chose none for all 16 choice sets and this participant enrolled in PrEP during the study.

### 3.3 PrEP product delivery DCE

**Figure 3:**
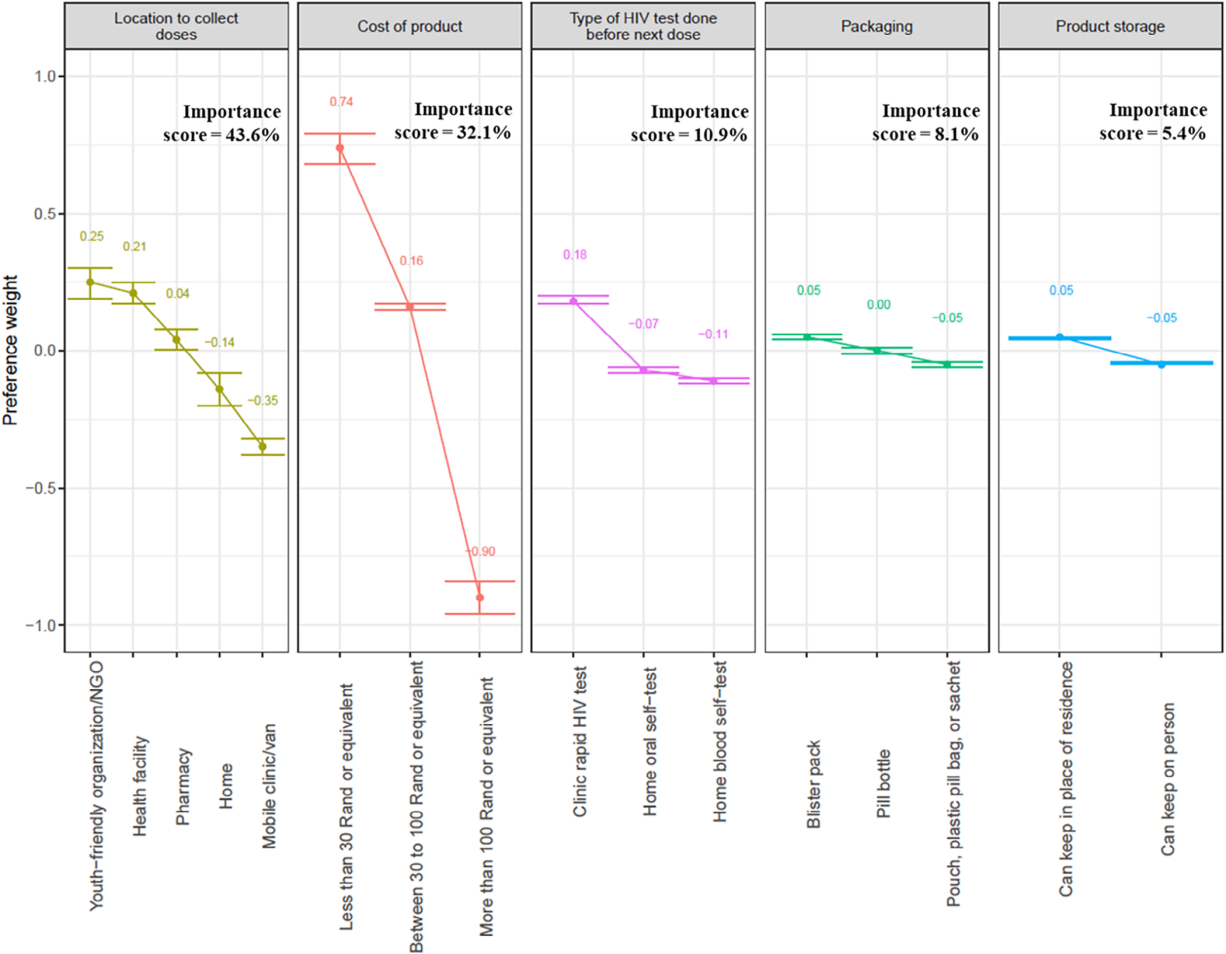
Month 3 preference weights with 95% confidence intervals. ^a^ The “cost of product” (August 2022 conversion rate), 30 Rand (South African rand) is approximately $2.00 and 100 Rand (South African rand) is approximately $6.00 United States dollars. NGO, non‐governmental organization.

At month 3, the biggest drivers of product delivery choices were attributes related to location to collect doses (importance score: 43.6%) and cost of product (importance score: 32.1%, Figure 3, Table S2, Figure S2). Regarding location to collect doses, a youth‐friendly organization/non‐governmental organization (NGO) was most preferred (PW: 0.25, 95% CI: 0.19, 0.30), followed by a health facility, whereas a mobile clinic/van was least preferred (Table S2). Regarding cost of product, less than R30 (∼ $1.50) was most preferred (PW: 0.74, 95% CI: 0.68, 0.79) and more than R100 (∼ $5.30) was least preferred (PW: −0.90, 95% CI: −0.96, −0.84). The type of HIV test done before the next dose was the third most important attribute (importance score: 10.9%), with clinic‐based rapid HIV test being the most preferred and a home‐based blood self‐test least preferred. For the month 3 DCE, 4% selected none (i.e. “I wouldn't choose either of these options”).

For both PrEP product and delivery DCEs, results were similar in the younger and older age groups (≤21 vs. >21 years, Tables S3 and S4, Figures S3 and S4). Stratified analyses of South Africa versus non‐South Africa sites (Tables S5 and S6, Figures S5 and S6) showed results largely consistent with the overall findings. However, in month 1, non‐South Africa sites most preferred a 2 kg weight gain (PW: 0.43, 95% CI: 0.38, 0.48), while in South Africa, the highest preference was for no weight change. For the “location to collect doses” attribute, non‐South African participants preferred most a health facility, whereas South African participants most preferred a youth‐friendly organization.

### 3.4 PrEP product and PrEP delivery attributes trade‐offs

Product form and dosing was the most important product attribute and, because most participants were taking daily oral PrEP, this trade‐off analysis focused on the relationship between this attribute and the weight change attribute which was the second most important (Table 2). For product form and dosing, there was utility loss from taking a large oral pill daily instead of a small oral pill monthly (1.16 at enrolment and 1.26 at month 1) or from taking a large oral pill daily instead of an injection every 6 months (0.73 at enrolment and 1.41 at month 1). Utility gain from a product with no weight change instead of a 5 kg weight loss was 1.29 at enrolment and 1.38 at month 1. Women would be willing to take a large oral pill daily instead of a small oral pill monthly or a large oral pill daily instead of an injection every 6 months if they would experience no weight change instead of a 5 kg weight loss with the other two products.

**Table 2:** Trade‐offs^a^ women were willing to make between pairs of attributes for PrEP product and delivery characteristics at enrolment, months 1 and 3, with 95% confidence intervals, INSIGHT cohort^b^, 2024

	PrEP product attributes				
	Product form and dosing		Weight change		
	Large oral pill daily	Large oral pill daily	No weight change	No weight change	No weight change
	Small oral pill monthly	Injection every 6 months	5 kg weight loss	2 kg weight loss	2 kg weight gain
**Enrolment**	−1.16 (−1.30, −1.03)	−0.73 (−0.85, −0.60)	1.29 (1.24, 1.35)	0.71 (0.67, 0.74)	0.36 (0.32, 0.40)
**Month 1**	−1.26 (−1.41, −1.11)	−1.41 (−1.56, −1.27)	1.38 (1.32, 1.44)	0.68 (0.64, 0.71)	0.38 (0.34, 0.42)

	PrEP delivery attributes				
	Cost of product^c^		Location to collect doses		
	More than 100 Rand or equivalent	More than 100 Rand or equivalent	Health facility	Youth‐friendly organization/NGO	Youth‐friendly organization/NGO
	Less than 30 Rand or equivalent	Between 30 and 100 Rand or equivalent	Mobile clinic or van	Mobile clinic or van	Health facility
**Month 3**	−1.64 (−1.72, −1.55)	−1.06 (−1.12, −1.00)	0.56 (0.51, 0.61)	0.60 (0.54, 0.66)	0.04 (−0.03, 0.11)

Abbreviations: kg, kilograms; NGO, non‐governmental organization; PrEP, pre‐exposure prophylaxis.

^a^ The difference in preference weights (utility loss or gain) between pairs of attribute levels is compared across various attributes. For example, the preference weight difference between a large oral pill daily and a small oral pill monthly (product form and dosing attribute) is compared to the difference between preference weights for no weight change and 5 kg weight loss (weight change attribute). Then, a high numeric difference indicated the participants’ willingness to choose one product attribute over the other one with a low numeric difference.

^b^ INSIGHT: Insights to advance PrEP discovery and delivery for African women.

^c^ The “cost of product” (August 2022 conversion rate), 30 Rand (South African rand) is approximately $2.00 and 100 Rand (South African rand) is approximately $6.00 United States dollars.

We considered PrEP delivery trade‐offs between the location to collect doses and cost of product attributes, given their relative importance (Table 2). Collecting doses from a health facility instead of a mobile clinic/van yielded a utility gain of 0.56. Collecting doses from a youth‐friendly organization/NGO instead of a mobile clinic/van yielded a gain of 0.60. There was a utility loss of 1.64 if the cost of the product was R100 or equivalent instead of less than R30 or equivalent. This indicates that if participants could collect their doses from a youth‐friendly organization/NGO or health facility instead of a mobile clinic/van, the women would be willing to pay R100 instead of <R30.

## 4 DISCUSSION

Our DCEs with almost 3000 young African women revealed strong preferences for longer‐acting oral and injectable formulations at baseline and after a month of oral PrEP use and for PrEP agents that did not change body weight. Of five PrEP delivery attributes, the location to collect doses was the most important with a strong preference for PrEP delivered at a youth‐friendly organization/NGO. Collectively, these findings highlight the most salient PrEP product options and delivery characteristics among young women, which may lead to greater PrEP coverage and reduce HIV incidence in this population.

In prior cross‐sectional stated‐preference studies conducted in South Africa [11, 12], women similarly indicated a strong preference for less frequent dosing and injectable formulations. Our findings suggest that as participants became familiar with oral PrEP, they had a greater preference for less frequently dosed and more discreet PrEP formulations. Young women may prefer discreet HIV prevention products to minimize the risk of unintentional disclosure and stigma [29] or to increase convenience. Participants initially preferred the 1‐month pill which was familiar as a pill but smaller than daily oral tenofovir‐based PrEP. However, this pill choice would still involve possibly monthly visits, with time and transport needed to reach the clinic, and young women may have preferred the 6‐month injection after experiencing regular pill‐taking, storage and clinic visits. Our results corroborate the findings of the Tablets, Ring, Injections as Options (TRIO) study, in which 62% of South African and Kenyan participants ranked injections as their first choice [30]. Our findings are also consistent with the HPTN 084 open‐label extension trial in which 78% of women chose bimonthly cabotegravir long‐acting (CAB‐LA) injections rather than oral PrEP [31, 32]. The MTN‐034/REACH trial also highlights young African women's preference for discreet and longer‐acting PrEP formulations, such as the monthly dapivirine ring, which aligns with our DCE results. In this randomized, open‐label crossover trial assessing the choice of the monthly dapivirine vaginal ring or daily oral PrEP among young women in South Africa, Uganda and Zimbabwe, similar proportions of young women preferred the ring to oral PrEP at enrolment [33], but after 6 months of use of each product, 65% of participants chose the ring and 30% chose oral PrEP [33]. Using objective adherence measures, MTN‐034/REACH found higher adherence, 57%, to both oral PrEP and dapivirine ring than had been previously observed [33]. This finding emphasizes the potential for improved adherence and persistence with PrEP when allowing individuals to choose options that maximize their preferences. While HPTN 084 and MTN‐034/REACH indicate that although most young women prefer a long‐acting PrEP product, findings also show a substantial minority will still choose daily oral PrEP.

Offering young women a choice of short and longer‐acting PrEP products is an ongoing priority for HIV prevention as new methods (e.g. lenacapavir) become available. Our results underscore the need for a choice of long‐acting formulations to meet the preferences of young African women, including those with dose forgiveness around clinic visit appointments. The findings also suggest that products that do not impact body weight are of great importance to women when considering a new PrEP product, which may reflect their concerns about the potential attribution of weight loss to illness or HIV. The latter finding aligns with our trade‐off analysis, which showed that this cohort of women would be willing to forgo their preferred PrEP product formulation if they could experience no weight change.

Delivery of PrEP in more community‐based settings could impact PrEP uptake and persistence among young African women. Preferences around PrEP delivery location (e.g. a youth‐friendly organization/NGO) have been rarely explored in DCEs with young women. One preference study among female sex workers in Malawi identified NGO‐run drop‐in centres as one of the most preferred means of receiving PrEP [14]. Other studies have indicated a preference for clinic‐based PrEP delivery [8, 11, 34], in part because of associated lower costs and higher perceived privacy compared to other locations [33]. Although cost is an important driving factor in PrEP implementation for young women, our trade‐off analysis indicated that women would give up their preferred cost if they could collect doses from a youth‐friendly organization/NGO or health facility. These findings highlight the importance of diversifying PrEP delivery locations while ensuring non‐judgemental, confidential clinical services [35].

The strengths of this study include a large multi‐country sample and longitudinal assessment of PrEP product attributes pre‐and‐post 1‐month use of daily oral PrEP to observe changing preferences after women experienced taking the current PrEP formulation, a large tablet daily. Our study evaluated PrEP delivery attributes separately from product attributes which allowed for a focus on PrEP service preferences that was not driven by trade‐offs with formulations and dosing. The limitations of this study include that the stated preferences might not represent actual choices. In contrast to most DCEs, our DCE included choices of both a daily oral pill formulation as participants were already using as well as long‐acting oral and injectable PrEP, currently in clinical development. This mix of choices provides participants with current real‐world PrEP choices and upcoming PrEP formulations becoming available, as was done in the TRIO DCE [30]. Despite efforts to constrain the number of choice tasks given to each participant, 16 scenarios may have placed a large cognitive burden on participants. Although our DCE is unique in exploring drug reversibility and dose forgiveness attributes, the terminology used to describe these attributes and underlying concepts may not have been readily understood even with the use of visual aids in the DCEs. The distinction between the type of protection (antiretroviral or immune‐based mechanism) may also have been difficult for participants to fully grasp. A large proportion of the sample was South African, and therefore, it is likely that the estimated preferences are driven by the South African sample. However, stratified analysis of South Africa and non‐South Africa sites showed mostly consistent findings suggesting that the results can be reasonably generalized to the target population of young African women. The CBC/HB model is a robust approach for analysing heterogeneous populations; however, it may not have completely accounted for heterogeneity across sites.

## 5 CONCLUSIONS

We evaluated preferences in PrEP product and delivery attributes among young women in Southern and Eastern Africa. Product form and dosing and weight change attributes exerted the greatest influence on product choice, and location to collect doses and cost were the most influential attributes regarding PrEP delivery. Product formulation preferences changed as women gained experience with daily PrEP, with women first preferring a monthly small oral pill at enrolment and then preferring a 6‐monthly injectable PrEP by month 1. The women most preferred to collect PrEP at youth‐friendly organizations/NGOs. Discreet, long‐acting prevention methods for HIV prevention and diverse PrEP delivery options are needed to offer women a choice of PrEP products and increase PrEP coverage among young women in sub‐Saharan Africa.

## COMPETING INTERESTS

The authors declare no conflict of interest.

## AUTHORS’ CONTRIBUTIONS

CC: Funding acquisition. RH, CC, JV, MLK, BGM, VOO and SD‐M: Conceptualization, study design. MLK: Project administration. SP: Data curation and management. WWD: Data analysis with guidance from RH, CC and JV and wrote the original draft of the manuscript. PLK, PS, JS, NM, CL, HN‐B, ZZ, RPHP, NM, MS, LN, RC, KG, PM, AVH and PdP: Implementation and administration of INSIGHT study at each site. All authors: Writing, reviewing, editing and approved the final version.

## FUNDING

The study was funded by the Bill and Melinda Gates Foundation (grant number: INV‐004743).

## Supporting information




**Table S1**: Product preference weights and the 95% confidence intervals, by visit, INSIGHT cohort, 2024 (*N* = 2847)
**Table S2**: Month 3 product delivery preference weights and the 95% confidence intervals, INSIGHT cohort, 2024
**Table S3**: Product preference weights, by age group and visit, INSIGHT cohort, 2024
**Table S4**: Month 3 product delivery preference weights, by age group, INSIGHT cohort, 2024 (*N* = 2594)
**Table S5**: Product preference weights and the 95% confidence intervals, by country site and visit, INSIGHT cohort, 2024
**Table S6**: Month 3 product delivery preference weights and the 95% confidence intervals, by country site, INSIGHT cohort, 2024
**Figure S1**: Overall product attribute importance scores (%), by visit
**Figure S2**: Month 3 product delivery attributes importance scores (%)
**Figure S3**: Overall product attribute importance scores (%), by age group and visit
**Figure S4**: Month 3 product delivery attributes importance scores (%), by age group
**Figure S5**: Overall product attribute importance scores (%), by country site and visit
**Figure S6**: Month 3 product delivery attributes importance scores (%), by country site

## Data Availability

The data that support the findings of this study are available from the corresponding author upon reasonable request through icrc@uw.edu, which will be reviewed by a central publications and manuscript committee.
