# Supplementary material for: Preferences for HIV pre‐exposure prophylaxis formulations and delivery among young African women: results of a discrete choice experiment
Source: J Int AIDS Soc. 2025 Feb 16;28(2):e26422. doi: 10.1002/jia2.26422 (PMC11830565; doi:10.1002/jia2.26422)
Supplement: Supplementary file 1 — Table S1: Product preference weights and the 95% confidence intervals, by visit, INSIGHT cohort, 2024 (N = 2847) Table S2: Month 3 product delivery preference weights and the 95% confidence intervals, INSIGHT cohort, 2024 Table S3: Product preference weights, by age group and visit, INSIGHT cohort, 2024 Table S4: Month 3 product delivery preference weights, by age group, INSIGHT cohort, 2024 (N = 2594) Table S5: Product preference weights and the 95% confidence intervals, by country site and visit, INSIGHT cohort, 2024 Table S6: Month 3 product delivery preference weights and the 95% confidence intervals, by country site, INSIGHT cohort, 2024 Figure S1: Overall product attribute importance scores (%), by visit Figure S2: Month 3 product delivery attributes importance scores (%) Figure S3: Overall product attribute importance scores (%), by age group and visit Figure S4: Month 3 product delivery attributes importance scores (%), by age group Figure S5: Overall product attribute importance scores (%), by country site and visit Figure S6: Month 3 product delivery attributes importance scores (%), by country site [file JIA2-28-e26422-s001.docx]

**Preferences for HIV Pre-Exposure Prophylaxis Formulations and Delivery among Young African Women – Results of a Discrete Choice Experiment**

**Supplemental Table 1: Product preference weights and the 95% confidence intervals, by visit, INSIGHT cohort^a^, 2024 (N=2847)**

| Attributes and levels | Enrollment | Month 1 |
| --- | --- | --- |
| Product form and dosing |  |  |
| Large oral pill daily | -0.49 (-0.58, -0.39) | -0.86 (-0.96, -0.75) |
| Small oral pill monthly | **0.67 (0.58, 0.77)** | 0.40 (0.30, 0.51) |
| Injection every 2 months | -0.32 (-0.37, -0.27) | -0.25 (-0.29, -0.20) |
| Injection every 3 months | -0.10 (-0.16, -0.04) | 0.15 (0.08, 0.21) |
| Injection every 6 months | 0.24 (0.16, 0.32) | **0.56 (0.46, 0.65)** |
| Weight change |  |  |
| 5kg weight loss | -0.68 (-0.72, -0.63) | -0.75 (-0.80, -0.70) |
| 2kg weight loss | -0.09 (-0.12, -0.06) | -0.05 (-0.07, -0.02) |
| 2kg weight gain | 0.26 (0.23, 0.28) | 0.25 (0.22, 0.28) |
| 5kg weight gain | -0.10 (-0.15, -0.06) | -0.08 (-0.13, -0.03) |
| No weight change | **0.62 (0.59, 0.64)** | **0.63 (0.60, 0.66)** |
| Drug reversibility |  |  |
| 1 week | -0.32 (-0.34, -0.29) | -0.36 (-0.41, -0.31) |
| 1 month | -0.10 (-0.12, -0.08) | -0.20 (-0.25, -0.15) |
| 6 months | 0.12 (0.10, 0.14) | 0.19 (0.15, 0.24) |
| 12 months | **0.29 (0.27, 0.32)** | **0.37 (0.31, 0.42)** |
| Dose forgiveness |  |  |
| 1 week | -0.17 (-0.18, -0.16) | -0.32 (-0.35, -0.30) |
| 2 weeks | -0.09 (-0.10, -0.08) | -0.25 (-0.27, -0.23) |
| 2 months | -0.06 (-0.07, -0.05) | 0.04 (0.03, 0.05) |
| 4 months | 0.13 (0.12, 0.14) | 0.18 (0.16, 0.20) |
| 7 months | **0.20 (0.18, 0.21)** | **0.35 (0.33, 0.37)** |
| Protection type |  |  |
| Medication | -0.02 (-0.03, -0.01) | -0.01 (-0.02, 0.004) |
| Immune | **0.02 (0.01, 0.03)** | **0.01 (-0.004, 0.02)** |
| None | -5.01 (-5.14, -4.88) | -6.22 (-6.38, -6.06) |

**Abbreviations:** Kg=kilograms.

^a^ INSIGHT: Insights to advance PrEP discovery and delivery for African women

**Supplemental Figure 1: Overall product attribute importance scores (%)^a^, by visit**

^a^ Reported as a percent and calculated for each participant – the difference between the highest and lowest level preference weights in the attribute (attribute range), divided by the sum of all ranges and multiplied by 100.

**Supplemental Table 2: Month 3 product delivery preference weights and the 95% confidence intervals, INSIGHT cohort^a^, 2024**

| Attributes and levels | N=2594 |
| --- | --- |
| Location to collect doses |  |
| Health facility | 0.21 (0.17, 0.25) |
| Pharmacy | 0.04 (0.003, 0.08) |
| Mobile clinic or van | -0.35 (-0.38, -0.32) |
| Youth-friendly organization or NGO | **0.25 (0.19, 0.30)** |
| Home | -0.14 (-0.20, -0.08) |
| Cost of product^b^ |  |
| Less than 30 Rand or equivalent | **0.74 (0.68, 0.79)** |
| Between 30 to 100 Rand or equivalent | 0.16 (0.15, 0.17) |
| More than 100 Rand or equivalent | -0.90 (-0.96, -0.84) |
| Type of HIV test done before next dose |  |
| Home oral self-test | -0.07 (-0.08, -0.06) |
| Home blood self-test | -0.11 (-0.12, -0.10) |
| Clinic rapid HIV test | **0.18 (0.17, 0.20)** |
| Packaging |  |
| Blister pack | **0.05 (0.04, 0.06)** |
| Pill bottle | 0.001 (-0.01, 0.01) |
| Pouch, plastic pill bag, or sachet | -0.05 (-0.06, -0.04) |
| Product storage |  |
| Can keep on person | -0.05 (-0.05, -0.04) |
| Can keep in place of residence | **0.05 (0.04, 0.05)** |
| None | -8.02 (-8.15, -7.89) |

**Abbreviations:** NGO=non-governmental organization.

^a^ INSIGHT: Insights to advance PrEP discovery and delivery for African women

^b^ The “cost of product” (August 2022 conversion rate), 30 Rand (South African rand) is approximately $2.00 and 100 Rand (South African rand) is approximately $6.00 United States dollars.

^b^

**Supplemental Figure 2: Month 3 product delivery attributes importance scores (%)^a^**

^a^ Reported as a percent and calculated for each participant – the difference between the highest and lowest level preference weights in the attribute (attribute range), divided by the sum of all ranges and multiplied by 100.

^b^ The “cost of product” (August 2022 conversion rate), 30 Rand (South African rand) is approximately $2.00 and 100 Rand (South African rand) is approximately $6.00 United States dollars.

**Supplemental Table 3: Product preference weights, by age group and visit, INSIGHT cohort^a^, 2024**

|  | 16-21 years (n=858) | | 22+ years (n=1989) | |
| --- | --- | --- | --- | --- |
| Attributes and levels | **Enrollment** | **Month 1** | **Enrollment** | **Month 1** |
| Product form and dosing |  |  |  |  |
| Large oral pill daily | -0.21 (-0.40, -0.02) | -0.30 (-0.51, -0.10) | -0.59 (-0.71, -0.46) | -0.93 (-1.09, -0.78) |
| Small oral pill monthly | **1.15** (**0.95, 1.34)** | **0.83** (**0.63, 1.03)** | **0.57** (**0.46, 0.69)** | 0.40 (0.26, 0.54) |
| Injection every 2 months | -0.46 (-0.56, -0.35) | -0.49 (-0.59, -0.39) | -0.33 (-0.39, -0.26) | -0.27 (-0.34, -0.20) |
| Injection every 3 months | -0.33 (-0.45, -0.21) | -0.18 (-0.31, -0.04) | -0.06 (-0.13, 0.02) | 0.15 (0.06, 0.24) |
| Injection every 6 months | -0.15 (-0.30, -0.002) | 0.13 (-0.05, 0.32) | 0.40 (0.30, 0.50) | **0.66** (**0.52, 0.79)** |
| Weight change |  |  |  |  |
| 5kg weight loss | -0.85 (-0.93, -0.76) | -1.10 (-1.21, -0.99) | -0.62 (-0.68, -0.57) | -0.60 (-0.66, -0.54) |
| 2kg weight loss | -0.24 (-0.30, -0.19) | -0.18 (-0.24, -0.11) | -0.04 (-0.07, -0.01) | -0.01 (-0.04, 0.02) |
| 2kg weight gain | 0.37 (0.31, 0.42) | 0.45 (0.39, 0.51) | 0.23 (0.19, 0.26) | 0.20 (0.17, 0.24) |
| 5kg weight gain | 0.03 (-0.06, 0.12) | 0.02 (-0.09, 0.14) | -0.17 (-0.23, -0.11) | -0.15 (-0.20, -0.09) |
| No weight change | **0.69** (**0.64, 0.75)** | **0.80** (**0.74, 0.87)** | **0.61 (0.58, 0.63)** | **0.56 (0.53, 0.59)** |
| Drug reversibility |  |  |  |  |
| 1 week | -0.34 (-0.40, -0.28) | -0.45 (-0.54, -0.37) | -0.40 (-0.42, -0.37) | -0.50 (-0.55, -0.45) |
| 1 month | -0.22 (-0.26, -0.18) | -0.29 (-0.38, -0.21) | -0.14 (-0.16, -0.12) | -0.30 (-0.34, -0.25) |
| 6 months | 0.20 (0.16, 0.23) | 0.32 (0.24, 0.40) | 0.18 (0.17, 0.20) | 0.29 (0.25, 0.33) |
| 12 months | **0.36** (**0.30, 0.42)** | **0.42 (0.33, 0.52)** | **0.35** (**0.32, 0.38)** | **0.51 (0.45, 0.56)** |
| Dose forgiveness |  |  |  |  |
| 1 week | 0.04 (0.02, 0.07) | -0.31 (-0.36, -0.27) | -0.22 (-0.23, -0.20) | -0.36 (-0.39, -0.33) |
| 2 weeks | **0.10** (**0.07, 0.13)** | -0.43 (-0.49, -0.37) | -0.12 (-0.14, -0.11) | -0.15 (-0.17, -0.13) |
| 2 months | -0.16 (-0.18, -0.14) | 0.02 (-0.01, 0.05) | -0.06 (-0.07, -0.05) | 0.03 (0.02, 0.04) |
| 4 months | -0.03 (-0.04, 0.01) | 0.32 (0.28, 0.37) | 0.15 (0.14, 0.17) | 0.15 (0.12, 0.17) |
| 7 months | 0.05 (0.02, 0.08) | **0.40** (**0.35, 0.46)** | **0.24 (0.23, 0.26)** | **0.34** (**0.31, 0.37)** |
| Protection type |  |  |  |  |
| Medication | -0.01 (-0.03, 0.01) | -0.02 (-0.04, 0.0003) | -0.03 (-0.04, -0.01) | -0.01 (-0.03, 0.004) |
| Immune | **0.01** (**-0.01, 0.03)** | **0.02** (**-0.0003, 0.04)** | **0.03** (**0.01, 0.04)** | **0.01 (-0.004, 0.03)** |
| None | -4.31 (-4.53, -4.08) | -5.40 (-5.70, -5.11) | -5.40 (-5.56, -5.23) | -6.49 (-6.69, -6.30) |

**Abbreviations:** Kg=kilograms.

^a^ INSIGHT: Insights to advance PrEP discovery and delivery for African women

**Supplemental Figure 3: Overall product attribute importance scores (%)^a^, by age group and visit**

^a^ Reported as a percent and calculated for each participant – the difference between the highest and lowest level preference weights in the attribute (attribute range), divided by the sum of all ranges and multiplied by 100.

**Supplemental Table 4: Month 3 product delivery preference weights, by age group, INSIGHT cohort^a^, 2024 (N=2594)**

| Attributes and levels | 16-21 years  (n=775) | 22+ years  (n=1819) |
| --- | --- | --- |
| Location to collect doses |  |  |
| Health facility | 0.22 (0.16, 0.29) | 0.21 (0.15, 0.26) |
| Pharmacy | 0.19 (0.12, 0.27) | -0.02 (-0.06, 0.02) |
| Mobile clinic or van | -0.43 (-0.49, -0.36) | -0.33 (-0.37, -0.29) |
| Youth-friendly organization or NGO | **0.36 (0.24, 0.48)** | **0.21 (0.15, 0.27)** |
| Home | -0.35 (-0.46, -0.23) | -0.06 (-0.13, 0.01) |
| Cost of product^b^ |  |  |
| Less than 30 Rand or equivalent | **0.73 (0.62, 0.83)** | **0.76 (0.69, 0.83)** |
| Between 30 to 100 Rand or equivalent | 0.10 (0.07, 0.12) | 0.20 (0.18, 0.21) |
| More than 100 Rand or equivalent | -0.82 (-0.93, -0.71) | -0.96 (-1.03, -0.88) |
| Type of HIV test done before next dose |  |  |
| Home oral self-test | -0.06 (-0.09, -0.04) | -0.08 (-0.09, -0.07) |
| Home blood self-test | -0.15 (-0.18, -0.13) | -0.09 (-0.11, -0.08) |
| Clinic rapid HIV test | **0.22 (0.18, 0.26)** | **0.17 (0.15, 0.19)** |
| Packaging |  |  |
| Blister pack | **0.04** (**0.02, 0.06)** | **0.06** (**0.05, 0.07)** |
| Pill bottle | -0.01 (-0.03, 0.02) | -0.01 (-0.02, 0.01) |
| Pouch, plastic pill bag, or sachet | -0.04 (-0.06, -0.02) | -0.05 (-0.06, -0.04) |
| Product storage |  |  |
| Can keep on person | -0.05 (-0.07, -0.03) | -0.05 (-0.06, -0.03) |
| Can keep in place of residence | **0.05 (0.03, 0.07)** | **0.05 (0.03, 0.06)** |
| None | -7.03 (-7.26, -6.79) | -8.26 (-8.42, -8.11) |

**Abbreviations:** NGO=non-governmental organization.

^a^ INSIGHT: Insights to advance PrEP discovery and delivery for African women

^b^ The “cost of product” (August 2022 conversion rate), 30 Rand (South African rand) is approximately $2.00 and 100 Rand (South African rand) is approximately $6.00 United States dollars.

^b^

**Supplemental Figure 4: Month 3 product delivery attributes importance scores (%)^a^, by age group**

^a^ Reported as a percent and calculated for each participant – the difference between the highest and lowest level preference weights in the attribute (attribute range), divided by the sum of all ranges and multiplied by 100.

^b^ The “cost of product” (August 2022 conversion rate), 30 Rand (South African rand) is approximately $2.00 and 100 Rand (South African rand) is approximately $6.00 United States dollars.

**Supplemental Table 5: Product preference weights and the 95% confidence intervals, by country site and visit, INSIGHT cohort^a^, 2024**

|  | South Africa (n=2120) | | Non-South Africa^b^ (n=727) | |
| --- | --- | --- | --- | --- |
| Attributes and levels | **Enrollment** | **Month 1** | **Enrollment** | **Month 1** |
| Product form and dosing |  |  |  |  |
| Large oral pill daily | -0.38 (-0.50, -0.26) | -0.81 (-0.97, -0.66) | -0.64 (-0.84, -0.44) | -1.32 (-1.56, -1.08) |
| Small oral pill monthly | **0.71** (**0.59, 0.83)** | 0.48 (0.32, 0.63) | **0.79 (0.65, 0.93)** | -0.02 (-0.18, 0.14) |
| Injection every 2 months | -0.45 (-0.52, -0.39) | -0.37 (-0.44, -0.30) | -0.21 (-0.30, -0.13) | 0.05 (-0.03, 0.13) |
| Injection every 3 months | -0.10 (-0.18, -0.02) | 0.15 (0.05, 0.24) | -0.19 (-0.30, -0.08) | 0.40 (0.26, 0.54) |
| Injection every 6 months | 0.23 (0.13, 0.33) | **0.56** (**0.42, 0.70)** | 0.26 (0.12, 0.39) | **0.89 (0.71, 1.08)** |
| Weight change |  |  |  |  |
| 5kg weight loss | -0.64 (-0.70, -0.58) | -0.72 (-0.78, -0.66) | -0.81 (-0.89, -0.73) | -0.82 (-0.91, -0.73) |
| 2kg weight loss | -0.06 (-0.09, -0.02) | 0.01 (-0.02, 0.04) | -0.24 (-0.30, -0.19) | -0.30 (-0.36, -0.24) |
| 2kg weight gain | 0.24 (0.21, 0.28) | 0.23 (0.19, 0.26) | 0.39 (0.35, 0.44) | **0.43 (****0.38, 0.48)** |
| 5kg weight gain | -0.22 (-0.27, -0.16) | -0.28 (-0.34, -0.22) | 0.17 (0.07, 0.27) | 0.34 (0.24, 0.45) |
| No weight change | **0.67 (0.64, 0.70)** | **0.76** (**0.72, 0.79)** | **0.49 (0.46, 0.52)** | 0.35 (0.30, 0.40) |
| Drug reversibility |  |  |  |  |
| 1 week | -0.45 (-0.48, -0.42) | -0.33 (-0.38, -0.27) | -0.16 (-0.20, -0.13) | -0.27 (-0.31, -0.22) |
| 1 month | -0.23 (-0.26, -0.21) | -0.18 (-0.24, -0.12) | 0.03 (-0.001, 0.06) | -0.06 (-0.09, -0.02) |
| 6 months | 0.25 (0.22, 0.27) | 0.15 (0.09, 0.21) | 0.003 (-0.02, 0.03) | 0.04 (0.01, 0.07) |
| 12 months | **0.44 (0.41, 0.47)** | **0.36 (0.30, 0.42)** | **0.13 (0.09, 0.18)** | **0.28 (0.23, 0.33)** |
| Dose forgiveness |  |  |  |  |
| 1 week | -0.15 (-0.17, -0.14) | -0.30 (-0.33, -0.27) | -0.19 (-0.22, -0.16) | -0.41 (-0.46, -0.36) |
| 2 weeks | -0.09 (-0.10, -0.07) | -0.25 (-0.28, -0.22) | -0.03 (-0.06, 0.004) | -0.12 (-0.15, -0.08) |
| 2 months | 0.001 (-0.01, 0.01) | 0.11 (0.10, 0.12) | -0.21 (-0.24, -0.19) | -0.06 (-0.08, -0.03) |
| 4 months | 0.09 (0.07, 0.10) | 0.16 (0.13, 0.18) | 0.18 (0.16, 0.21) | 0.17 (0.14, 0.19) |
| 7 months | **0.16** (**0.14, 0.17)** | **0.29 (0.26, 0.31)** | **0.25 (0.21, 0.29)** | **0.42** (**0.37, 0.47)** |
| Protection type |  |  |  |  |
| Medication | -0.01 (-0.03, -0.002) | -0.02 (-0.04, -0.01) | -0.04 (-0.07, -0.01) | -0.01 (-0.04, 0.03) |
| Immune | **0.01 (0.002, 0.03)** | **0.02 (0.01, 0.04)** | **0.04 (0.01, 0.07)** | **0.01 (-0.03, 0.04)** |
| None | -4.89 (-5.05, -4.73) | -6.14 (-6.35, -5.93) | -5.40 (-5.63, -5.16) | -6.22 (-6.46, -5.99) |

**Abbreviations:** Kg=kilograms.

^a^ INSIGHT: Insights to advance PrEP discovery and delivery for African women

^b^ Non-South Africa sites: Eswatini, Kenya, Malawi Uganda, and Zambia.

^b^

**Supplemental Figure 5: Overall product attribute importance scores (%)^a^, by country site and visit**

^a^ Reported as a percent and calculated for each participant – the difference between the highest and lowest level preference weights in the attribute (attribute range), divided by the sum of all ranges and multiplied by 100.

^b^ Non-South Africa sites: Eswatini, Kenya, Malawi Uganda, and Zambia.

**Supplemental Table 6: Month 3 product delivery preference weights and the 95% confidence intervals, by country site, INSIGHT cohort^a^, 2024**

| Attributes and levels | South Africa sites (n=1894) | Non-South Africa^b^  (n=700) |
| --- | --- | --- |
| Location to collect doses |  |  |
| Health facility | -0.04 (-0.09, 0.02) | **0.77** (**0.70, 0.83)** |
| Pharmacy | -0.0003 (-0.05, 0.05) | 0.13 (0.09, 0.17) |
| Mobile clinic or van | -0.34 (-0.39, -0.30) | -0.44 (-0.48, -0.39) |
| Youth-friendly organization or NGO | **0.32** (**0.24, 0.40)** | 0.13 (0.09, 0.18) |
| Home | 0.06 (-0.02, 0.15) | -0.59 (-0.66, -0.53) |
| Cost of product^c^ |  |  |
| Less than 30 Rand or equivalent | **0.81 (0.73, 0.90)** | **0.65** (**0.57, 0.72** |
| Between 30 to 100 Rand or equivalent | 0.20 (0.19, 0.21) | 0.11 (0.09, 0.13) |
| More than 100 Rand or equivalent | -1.01 (-1.10, -0.93) | -0.76 (-0.84, -0.67) |
| Type of HIV test done before next dose |  |  |
| Home oral self-test | -0.06 (-0.07, -0.05) | -0.13 (-0.16, -0.10) |
| Home blood self-test | -0.10 (-0.11, -0.08) | -0.14 (-0.16, -0.11) |
| Clinic rapid HIV test | **0.16 (0.14, 0.18)** | **0.27** (**0.23, 0.31)** |
| Packaging |  |  |
| Blister pack | **0.05** (**0.04, 0.06)** | **0.05** (**0.03, 0.07)** |
| Pill bottle | -0.02 (-0.03, -0.002) | 0.02 (-0.01, 0.05) |
| Pouch, plastic pill bag, or sachet | -0.03 (-0.05, -0.02) | -0.07 (-0.09, -0.04) |
| Product storage |  |  |
| Can keep on person | -0.01 (-0.02, -0.003) | -0.13 (-0.16, -0.11) |
| Can keep in place of residence | **0.01** (**0.003, 0.02)** | **0.13 (0.11, 0.16)** |
| None | -8.92 (-9.10, -8.75) | -6.54 (-6.72, -6.36) |

**Abbreviations:** NGO=non-governmental organization.

^a^ INSIGHT: Insights to advance PrEP discovery and delivery for African women

^b^ Non-South Africa sites: Eswatini, Kenya, Malawi Uganda, and Zambia.

^c^ The “cost of product” (August 2022 conversion rate), 30 Rand (South African rand) is approximately $2.00 and 100 Rand (South African rand) is approximately $6.00 United States dollars.

^b^

**Supplemental Figure 6: Month 3 product delivery attributes importance scores (%)^a^, by country site**

^a^ Reported as a percent and calculated for each participant – the difference between the highest and lowest level preference weights in the attribute (attribute range), divided by the sum of all ranges and multiplied by 100.

^b^ The “cost of product” (August 2022 conversion rate), 30 Rand (South African rand) is approximately $2.00 and 100 Rand (South African rand) is approximately $6.00 United States dollars.
